# Dietary diversity and opportunistic infections among adults living with human immunodeficiency virus on antiretroviral therapy in Kumasi metropolis; a facility-based cross-sectional study

**DOI:** 10.1186/s12879-024-10395-z

**Published:** 2025-01-02

**Authors:** Charles Apprey, Hammond Yaw Addae, Monica Osei, Irene Danquah, Reginald Annan

**Affiliations:** 1 Department of Biochemistry, College of Science, KNUST, Kumasi, Ghana; 2 Nursing & Midwifery Training College, Kpembe, Ghana; 3 Olime Health, Accra, Ghana

**Keywords:** Antiretroviral therapy, CD4 count, Dietary diversity, Ghana, Malnutrition, Opportunistic infections, People living with HIV/AIDS

## Abstract

**Background:**

Despite advances in antiretroviral therapy (ART), people living with human immunodeficiency virus (HIV)/ acquired immunodeficiency syndrome (AIDS) continue to face heightened susceptibility to opportunistic infections (OIs). Adequate nutrition remains an essential factor that positively influences disease progression and the occurrence of OIs. In Ghana, no study has evaluated the association between dietary diversity and OI occurrence among adults with HIV. This study aimed to evaluate the association between dietary diversity and the presence of OIs among HIV-positive adults receiving ART.

**Methods:**

A facility-based cross-sectional study was conducted among 291 HIV-positive adults receiving ART from February 2023 to April 2023 at Kumasi South Hospital, Ghana. The study participants were selected using a convenient sampling method. A pre-tested questionnaire and review of electronic health records were used to collect sociodemographic, nutritional and clinical data. Binary logistic regression analyses were conducted to identify variables significantly associated with the study outcome and hierarchical multivariable logistic regression was used to evaluate the association between dietary diversity and the occurrence of OIs while controlling for confounders at p-value < 0.05.

**Results:**

The mean age and dietary diversity were 46.2 ± 10.9 years and 4.0 (IQR: 3.0 to 6.0) food groups, respectively. Out of 291 respondents, 152 (52.2%) had inadequate dietary diversity and 39 (13.4%) had at least one OI. The respondents with inadequate dietary diversity were three times more likely to have an OI than their peers with adequate dietary diversity [AOR 3.03, (95% CI: 1.20 to 7.64), *p* = 0.019].

**Conclusion:**

This study revealed that inadequate dietary diversity is a significant nutritional problem and dietary diversity was associated with the presence of OIs among PLWHA on ART at the study site. Hence, there is the need to enhance the intake of diversified diets based on locally available foods. This could decrease the occurrence of OIs and eventually reduce HIV-related morbidity/mortality.

**Supplementary Information:**

The online version contains supplementary material available at 10.1186/s12879-024-10395-z.

## Introduction

Human immunodeficiency virus (HIV)/ acquired immunodeficiency syndrome (AIDS) remains a significant global public health challenge, particularly in Sub-Saharan Africa, where it exerts significant impact on healthcare systems [1]. Despite advances in antiretroviral therapy (ART) and strides in HIV treatment, people living with HIV/AIDS (PLWHA) continue to face heightened susceptibility to opportunistic infections (OIs) occasioned by poor HIV-related immunosuppression [1, 2]. Globally, 39.9 million people are estimated to be living with HIV/AIDS, and 630,000 people died from AIDS-related illnesses in 2023 [3]. OIs have been reported to be the leading cause of hospitalization, accounting for a significant proportion of mortality in resource-poor settings [4, 5]. These OIs, arising from pathogens that typically do not cause illness in healthy individuals pose substantial risks to PLWHA, often leading to increased morbidity and mortality [6]. It has been established that these otherwise less harmful infections can thrive due to the weakened immune system associated with HIV/AIDS [7].

A myriad of factors, including adherence to ART [8], enhanced nutritional status [8, 9], psychosocial support in the form of reduction in stigmatization [10] and regular monitoring of CD4 counts [11] could help ameliorate the severity of OIs and HIV progression among PLWHA. Among these factors, nutrition and adherence to ART have been found to attenuate HIV progression [8]. Recent advances in HIV/AIDS research have improved the efficacy of ART [12] and it is anticipated that ART patients should not regularly experience OIs. However, in low-resource settings, PLWHA may face elevated risks of OIs due to the regularity of exposure to disease-causing organisms and low dietary diversity [5].

In Ghana, there are quite a number of studies on nutrition and OIs among PLWHA. Specifically, these studies have focused largely on nutrition and nutrition-related factors [13–19] or OIs and OI-related factors [20–23]. However, no study has assessed the association between nutrition (i.e. dietary diversity) and the presence of OIs among adults with HIV, necessitating the need to fill this knowledge gap.

Kumasi is the second most populous city in Ghana and the capital of the Ashanti Region. Together with Accra, Ghana’s capital, they are reported to have the largest number of PLWHA, with the Ashanti region accounting for 77,000 PLWHA as of 2020 [24]. The metropolis grapples with a significant HIV/AIDS burden and accompanying high rates of OIs despite efforts to expand access to ART, healthcare services and nutritional support. The availability and access to ART are intermittent, and nutritional support may be inadequate at some facilities [25]. Thus, the effects of OIs on PLWHA is quite significant. Therefore, it is important to ascertain how dietary diversity affects the occurrence of OIs, given the unique circumstances and paucity of literature.

This study aimed to examine the association between dietary diversity and OIs among adults on ART in a referral health facility in the Kumasi Metropolis of Ghana. Specifically, this study assessed the dietary intake and food habits of adults on ART, the prevalence of OIs and other clinical factors amongst adults on ART, and the association between dietary diversity and the presence of OIs among adults on ART. This study could contribute to the design of evidence-based interventions to foster improved nutrition and health outcomes amongst this vulnerable population.

## Methods

### Study design and duration

A health facility-based cross-sectional study was conducted among adults aged 18 years and above from 1st February 2023 to 30th April 2023. The participants were registered for support and care at the hospital’s ART clinic and were accessing ART services within the period of data collection.

### Study area

The study was carried out at the ART clinic in the Kumasi South Hospital of the Ashanti Region of Ghana. Kumasi metropolis is made up of 10 sub-metropolises that are responsible for administering healthcare. The metropolis has 79 health facilities, that include a teaching hospital, three government hospitals, 24 private hospitals, 44 clinics, four maternity homes and four health centres [26]. The Kumasi South Hospital has a bed capacity of about 140 with 6,660 admissions per year and serves an estimated catchment area population of 282,609 [27]. Clinical services provided by the hospital include general consultation, pharmacy, paediatrics, surgery, obstetrics, gynaecology, ophthalmology, ear nose and throat, laboratory, and an ART clinic. The ART clinic has a staff of 20 and has been operational since 2008. It provides services that include HIV testing and counselling, treatment and support for PLWHA, prevention of mother-to-child transmission, TB/HIV/co-infection diagnoses and management, differentiated service delivery across all entry points of the hospital, and comprehensive HIV prevention services such as distribution of condoms, and post-exposure prophylaxis. These services have been extended to include key population groups such as men having sex with men and female sex workers [28]. Additionally, the clinic conducts community and school outreaches to enhance sensitization. The ART clinic of the Kumasi South Hospital was purposively selected, considering that it is the main referral center serving a relatively large number of HIV patients in the Kumasi metropolis.

### Eligibility criteria

The study included HIV/AIDS-positive adults aged 18 years and above who resided in the Kumasi metropolis for a minimum of 6 months and received ART services at the Kumasi South Hospital. Only potential participants who consented to participate in the study were included. The study excluded pregnant women with HIV/AIDS, lactating mothers with HIV/AIDS and patients with HIV/AIDS who were critically ill at the time of data collection. Potential participants who were part of a nutritional intervention study and those on their second visit during the data collection period were excluded.

### Sample size calculation

The sample size of the study was calculated using Yamane’s sample size formula, which is based on a finite population size [29].

$$\:n=\:\frac{N}{1+N\:{\left(e\right)}^{2}}$$

The average attendance at the ART centre was 22 per day. The total attendance during the eight weeks (40 working days) period of data collection was 880. Given the estimated total population (N) of 880, 95% confidence interval, and 0.05 margin of error (e), the sample size (n) was calculated to be 273. To account for attrition and incomplete data, an additional 28 (10%) respondents were included giving a total sample size of 301.

### Sampling procedures

A convenient sampling procedure was used to select consenting participants in this study. Participants who met the inclusion criteria were recruited during the scheduled days for CD4 count test until the sample size was attained. The registration numbers of each client were recorded at the time of the interview to avoid possible repetition.

### Data collection procedures

Data was collected by four enumerators who were trained on the appropriate codes of ethics when interacting with participants to avoid any stigmatization. The nurse in charge of the ART clinic assisted in the orientation of the study enumerators before data collection. Prior to data collection, the questionnaire was pre-tested on 14 PLWHA from a different hospital representing 5% of the sample size. Based on responses from the pretest, the study instrument was corrected to resolve grammatical and skip pattern errors. All questionnaires were administered in the participants’ preferred language.

Electronic health records were reviewed to retrieve relevant medical data such as haemoglobin, current CD4 count, presence and type of OI as medically diagnosed. For a patient to be classified as having an OI in this study, the patient should have been diagnosed with OI within the period of the study data collection. A bioelectrical impedance analyser (Omron BF511) was used to assess the body composition (weight, body fat and visceral fat) of the study participants [30]. A stadiometer was used to measure the height of participants following standard procedures. To minimize random instrumental error, the instruments were calibrated each week, and all measurements were duplicated and the average calculated.

### Study instrument

Participants’ sociodemographic and economic characteristics, household food security, nutritional characteristics and dietary habits, and clinical factors were collected using an interviewer-administered structured questionnaire. The questionnaire included the following variables.

#### Sociodemographic variables

This included sex, age, education, employment and marital status. Income in terms of the nominal amount earned within a month was categorized in Ghanaian cedis as low (< 500), medium (from 500 to 2999) or high (≥ 3000).

#### Types of antiretroviral therapy

Medications for treating HIV/AIDS included Dolutegravir (DGT)-based, Efavirenz (EFV)-based and Nevirapine (NVP)-based regimens.

#### Antiretroviral therapy duration

The number of years each participant had been on ART [31].

#### Alcohol intake

This was assessed through self-reported consumption of alcoholic beverages in the past 7 days.

#### Body mass index (BMI)

BMI was calculated as weight in kilograms divided by height in meters squared (kg/m²) and categorized according to World Health Organization (WHO) guidelines, where respondents with BMI ≤ 18.5 kg/m² were considered underweight, those between 18.5 kg/m² and 25.0 kg/m² were considered normal weight, and those ≥ 25 kg/m² were considered overweight/obese [32].

#### CD4 count

The most recent CD4 count was obtained from medical records and treated as a categorical variable where a low CD4 count was < 500 cell/µl and a high CD4 count ≥ 500 cell/µl according to the classifications of the WHO [33].

#### Anaemia

Reports on anaemia were obtained from the medical records and classified as no anaemia (≥ 10.5 g/dL), grade I anaemia (9.5 to 10.49) g/dL, grade II anaemia (8.0 to 9.49) g/dL, grade III anaemia (6.5 to 7.9) g/dL or Grade IV anaemia (< 6.5 g/dL) on the basis of the patient haemoglobin concentration. This categorization was based on the WHO AIDS Clinical Trials Group (WHO/ACTG) anaemia grades [34]. The various grades of anaemia were subsequently categorized as anaemic (< 10.5 g/dL) and non-anaemic (≥ 10.5 g/dL).

#### Household food security

Household food insecurity was assessed using the Household Food Insecurity Access Scale (HFIAS) tool [35]. It assesses whether households have experienced difficulties accessing adequate food over the past 4 weeks. The HFIAS consists of nine questions that have been utilized in several countries and have effectively differentiated between food-secure and food-insecure households. For each question, respondents indicate how often the experience occurred; “Never” had a score of 0, “Rarely” (1 & 2 times) had a score of 1, “Sometimes” (3 to 10 times) had a score of 2, and “Often” (more than 10 times) had a score of 3. This gives a score range from 0 to 27, with greater scores indicating greater household food insecurity. Respondents with HFIAS scores below or equal to the mean (7.4) were deemed to have food-secured households; otherwise, they were categorized as food insecure.

#### Dietary diversity score (DDS)

The dietary diversity variable was assessed using the validated dietary diversity tool [36]. This tool was used in this study as a proxy measure of the nutritional quality of an individual’s diet for adult men and women similar to studies in North-Western Ghana and Latin America [17, 37].

The questionnaire containing the independent, dependent and covariates as administered to respondents in this study has been attached as **supplementary file 1**.

### Study variables

#### Dependent variable

The dependent variable was the presence of OIs. As recommended by the WHO and Ghana Health Service (GHS) / National AIDS Control Program (NACP) guidelines, the diagnosis of OIs was based first on assessing clinical manifestations and additional laboratory confirmatory tests or chest X-ray scans where necessary [33, 38]. For instance, in the case of tuberculosis, patients with symptoms like persistent cough (≥ 2 weeks), weight loss, night sweats, and fever were tested for tuberculosis. Sputum examination using microscopy were conducted to detect the presence of the causative organism e.g. *Mycobacterium tuberculosis* as a confirmatory laboratory test. Scans of chest X-ray were used as an alternate confirmatory test for patients suspected of having pulmonary tuberculosis. Sudden onset of fever, cough with sputum, shortness of breath, and chest pain were suggestive symptoms of patients with bacterial pneumonia. Chest X-ray was used to identify consolidation in the lungs and sputum, or blood cultures were conducted as confirmatory tests to identify causative organisms, which could include *Streptococcus pneumoniae*, *Haemophilus influenzae*, or *Staphylococcus aureus.* Assessment of clinical manifestation in addition to confirmatory tests were conducted for other OIs including esophageal candida, oral candida, chronic diarrhoea, cerebral toxoplasmosis or pneumocystis pneumonia [33, 38].

#### Independent variables

The dietary diversity score (DDS) was calculated on the basis of the consumption of food in 24 h from the 10-food group categorization: (1) grains, white roots and tubers, and plantains; (2) beans, peas and lentils; (3) nuts and seeds; (4) milk and milk products; (5) meat, poultry and fish; (6) eggs; (7) dark green leafy vegetables; (8) other vitamin A rich fruits and vegetables; (9) other vegetables; and (10) other fruits. The respondent was scored zero if they did not consume food from any food group or one if they did. In sum, each participant’s score ranged from 0 to 10, with higher scores indicating greater dietary diversity. The DDS was categorized into two groups: adequate dietary diversity (DDS ≥ 5) and inadequate dietary diversity (DDS < 5) based on FAO & FHI guidelines [39].

### Covariates

Variables were selected based on literature and included in this study for their potential associations with dietary diversity and the presence of OIs in PLWHA [9]. These included the socio-demographic and economic variables, types of ART, duration of ART, alcohol intake, BMI, CD4 count, anaemia status and household food security.

### Ethics approval and consent to participate

Ethical clearance was obtained from the Committee of Human Research, Publication, and Ethics, School of Medical Sciences, Kwame Nkrumah University of Science and Technology (CHRPE/AP/186/23). Additionally, approval was sought from the Health Directorate of the Kumasi South Hospital (KSH/RESH-50). Informed consent was obtained from all participants. The participants were educated on the relevance of the study, and their enrolment was voluntary. Participants were able to withdraw completely or halt their participation midway without any repercussions. All information provided to the interviewers were strictly confidential, and records were securely stored in a locker. Participants were given nutrition education, and the study outcome was communicated to them. This study was conducted in compliance with the Helsinki Declaration; Ethical Principles for medical research involving human subjects [40].

### Data analysis

All the statistical analyses were conducted using IBM SPSS software version 24. Descriptive statistics were performed to determine frequencies and percentages for categorical variables and means and standard deviations for continuous variables without outliers. Median and interquartile range (IQR) were used as measures of central tendency and spread for dietary diversity and haemoglobin variables. Bivariate and multivariable logistics regression analyses were used to investigate the associations between dietary diversity and the presence of OIs. In both instances, a binary coding was adopted where participants were classified as either OI positive or OI negative. At the bivariate level, significant variables at p-values < 0.05 were included in the multivariable analyses. Three models were developed to assess this relationship while adjusting for confounders. In the first model, the association between dietary diversity and OIs was examined without adjustments for additional covariates. This served as a baseline analysis to determine the crude relationship between the two variables. The second model incorporated age and income as covariates to account for confounding by sociodemographic factors. In the third and final model, further adjustments were made for nutritional and clinical factors. These included the number of years on ART, alcohol intake, household food security, BMI, anaemia status, and CD4 count. All the models were assessed for multicollinearity and goodness of fit using correlation matrix and the Wald test statistic, respectively. Statistical significance was set at a p-value of < 0.05, and 95% confidence intervals were calculated for all estimates.

## Results

### Background characteristics of HIV + patients receiving antiretroviral therapy

A total of 291 respondents were sampled and included in this study. The mean age of all the respondents was 46.2 ± 10.9 years, with 64.6% being middle-aged (36–55 years). Younger (18–35 years) and older (56 years or more) participants represented similar percentages of approximately 18% of the sample. A substantial majority (85.2%) of the respondents were female and employed (84.9%). Slightly more than half (55%) had monthly incomes ranging from GHS 500 to GHS 2,999. About 29.2% had high income (GHS 3,000 +), and 15.8% had low income (< GHS 550). Over one-third (35.7%) were married, and almost half were either widowed (22.7%), or divorced / separated (26.5%). The mean household size was 4.3 ± 2.7, with almost 3 in 5 having female household heads. The most common ART medication administered was DTG (84.5%). Majority (56.0%) of the respondents had been on ART for more than 5 years, whereas 17.2% and 26.8% have been on ART for < 1 year and between 1 and 5 years, respectively. In all, 13.4% of the respondents were diagnosed with OIs and 57.0% had low CD4 counts (< 500 cell/µl) Table 1.

**Table 1:** Background characteristics of HIV + patients on antiretroviral therapy in Kumasi South Hospital (*n* = 291)

Characteristic	Frequency (%) / mean ± standard deviation
**Mean age (years)**	46.2 ± 10.9
**Age (years)**	
18–35	52(17.9)
36–55	188(64.6)
56+	51(17.5)
**Gender**	
Male	43(14.8)
Female	248(85.2)
**Education**	
None	39(13.4)
Basic	180(61.9)
Junior high school	40(13.7)
Secondary +	32(11.0)
**Monthly income (Ghanaian cedis)**	
Low (< 550)	46(15.8)
Medium (550–2999)	160(55.0)
High (≥ 3000)	85(29.2)
**Marital status**	
Married	104(35.7)
Single	44(15.1)
Widowed	66(22.7)
Divorced / Separated	77(26.5)
**Religion**	
Christian	256(88.0)
Islam	31(10.7)
None	4(1.4)
**Employed?**	
No	44(15.1)
Yes	247(84.9)
**Ethnicity**	
Akan	203(69.8)
Ewe	14(4.8)
Ga-Adamgbe	2(0.7)
Mole-Dagbani	59(20.3)
Guan	13(4.5)
**Mean household size**	4.3 ± 2.7
**Gender of household head**	
Male	119(40.9)
Female	172(59.1)
**Mean years on antiretroviral therapy**	7.0 ± 5.5
**Number of years on antiretroviral therapy**	
≤ 1	50(17.2)
1 to 5	78(26.8)
> 5	163(56.0)
**Type of antiretroviral therapy**	
Dolutegravir (DGT)-based	246(84.5)
Efavirenz (EFV)-based	28(9.6)
Nevirapine (NVP)-based	17(5.8)
**Presence of opportunistic infection**	
No	252(86.60)
Yes	39(13.40)
**Mean CD4 count**	432.4 ± 188.4
**CD4 count**	
Low (< 500cell/µl)	166(57.0)
High (≥ 500cell/µl)	125(43.0)

**Fig. 1:**
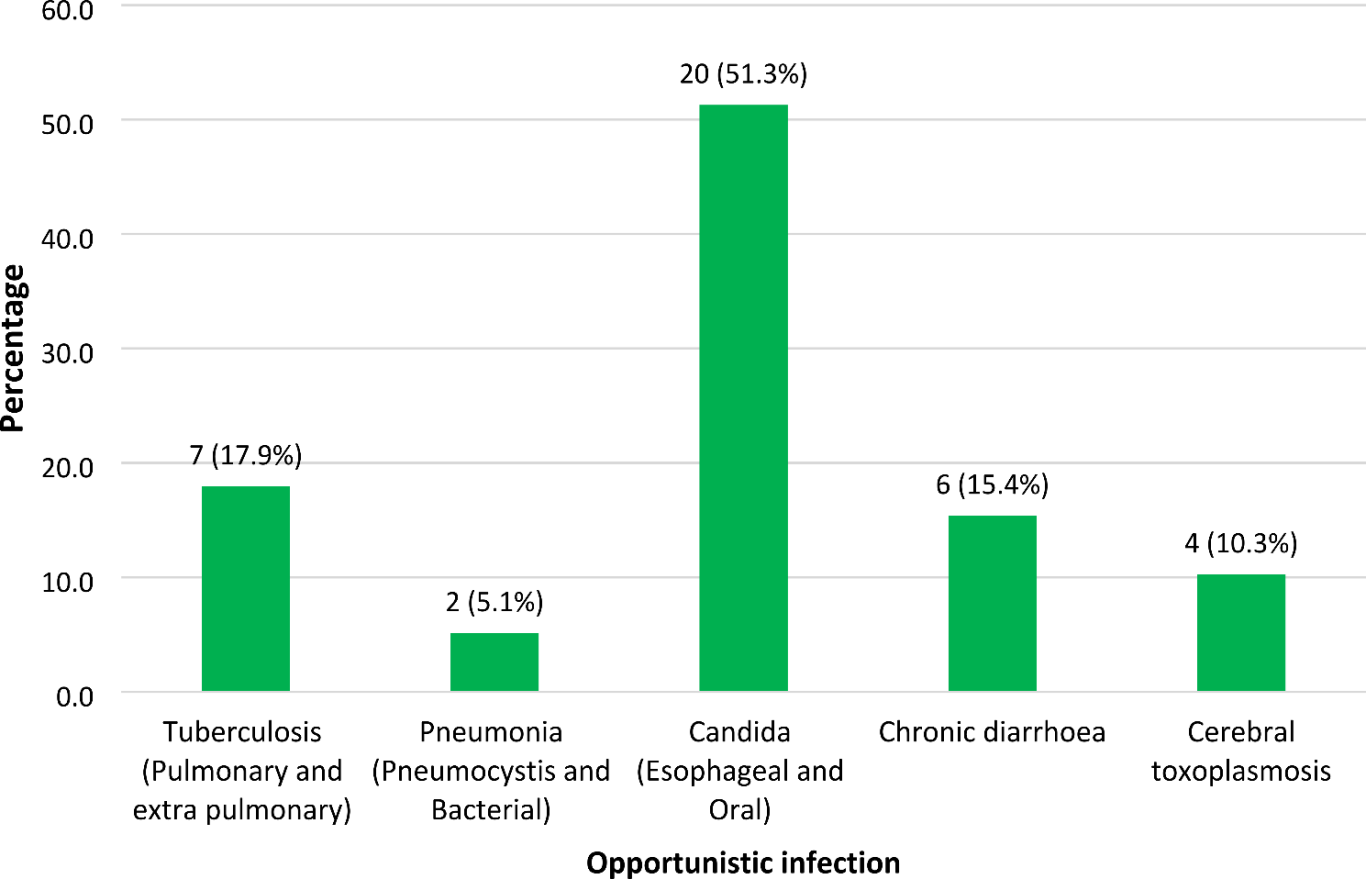
Frequencies of the types of opportunistic infections among HIV-positive adults receiving antiretroviral therapy (*n* = 39)

The most prevalent OIs were candida (esophageal and oral) and tuberculosis (pulmonary and extrapulmonary) with prevalence rates of 51.3% and 17.9% respectively. Additionally, the prevalence of chronic diarrhoea (15.4%), cerebral toxoplasmosis (10.3%) and pneumonia (pneumocystis and bacterial) (5.1%) was less than 20% (Fig. 1).

### Nutritional characteristics and food consumption patterns of HIV + adults

The median DDS was 4.0 (IQR: 3.0 to 6.0) food groups, with 47.8% meeting the minimum dietary diversity for adults (Table 2). Approximately 3 in 5 respondents (60.8%) were food insecure, whereas 2 in 5 were food secured. The mean BMI was 25.7 ± 5.5 kg/m^2^ with 40.9% being normal weight and 51.2% overweight or obese. The median haemoglobin level was 10.0 g/dL (IQR: 8.9 to 11.1) g/dL and 61.5% of the patients were anaemic. About 6 in 10 respondents (60.5%) had skipped a meal, and 14.8% had consumed alcohol in the past week. In the food groups, 96.2% and 77.7% of the respondents consumed foods from group 9 (other vegetables) and group 1 (grains, white roots and tubers), respectively. Group 10 (other fruits) had the lowest consumption of 8.2%. Notably, Group 4 (milk and milk products) and Group 5 (meat, poultry and fish) also had low consumption rates of 20.6% and 21.6%, respectively.

**Table 2:** Nutritional characteristics and food habits of HIV + adults on antiretroviral therapy

Characteristic	Frequency (%) / mean ± standard deviation /median (IQR)
**Mean dietary diversity score**	4.0 (IQR: 3.0 to 6.0)
**Adequate dietary diversity**	
No (< 5 food groups)	152(52.2)
Yes (> 4 food groups)	139(47.8)
**Mean household food insecurity**	7.4 ± 7.38
**Household food insecurity**	
Food secure	114(39.2)
Food insecure	117(60.8)
**Mean body mass index (kg/m** ^**2**^ **)**	25.7 ± 5.46
**Body Mass Index**	
Underweight (< 18.5 kg/m^2^)	23(7.9)
Normal (18.5 kg/m^2^ to 24.9 kg/m^2^)	119(40.9)
Overweight/obese (≥ 25 kg/m^2^)	149(51.2)
**Mean haemoglobin level (g/dL)**	10.0 (IQR: 8.9 to 11.1)
**Anaemic (Haemoglobin)**	
No (≥ 10.5 g/dL)	112(38.5)
Yes (< 10.5 g/dL)	179(61.5)
**Meals eaten (past 24 h)**	
Two times	84(28.9)
Three times	189(64.9)
Four times	18(6.2)
**Skipping meals (past week)**	
No	115(39.5)
Yes	176(60.5)
**Alcohol intake (past week)**	
No	248(85.2)
Yes	43(14.8)
**Percent body fat (%)**	
Not high	97(33.3)
High	194(66.7)
**Visceral fat (% body weight)**	
Normal (< 10%)	234(80.4)
High (10% +)	57(19.6)
**Group 1 (Grains**,** white roots and tubers)**	
No	65(22.3)
Yes	226(77.7)
**Group 2 (Beans**,** peas and lentils)**	
No	205(70.4)
Yes	86(29.6)
**Group 3 (Nuts and seeds)**	
No	246(84.5)
Yes	45(15.5)
**Group 4 (Milk and milk products)**	
No	231(79.4)
Yes	60(20.6)
**Group 5 (Meat**,** poultry and fish)**	
No	228(78.4)
Yes	63(21.6)
**Group 6 (Eggs)**	
No	210(72.2)
Yes	81(27.8)
**Group 7 (Dark green leafy vegetables)**	
No	189(64.9)
Yes	102(35.1)
**Group 8 (Vitamin A-rich fruits and vegetables)**	
No	225(77.3)
Yes	66(22.7)
**Group 9 (Other vegetables)**	
No	11(3.8)
Yes	280(96.2)
**Group 10 (Other fruits)**	
No	267(91.8)
Yes	24(8.2)

### Association between dietary diversity and the occurrence of opportunistic infections among HIV + adults on antiretroviral therapy

Table 3 shows that those with inadequate dietary diversity were three times more likely to have OIs compared with respondents with adequate dietary diversity and adjusting for confounding variables did not attenuate this significant association in model 3 [AOR 3.03, (95% CI: 1.20 to 7.64), *p* = 0.019)]. All three models were statistically significant according to the Wald test, with p-values < 0.05.

**Table 3:** Binary logistic regression analysis of the association between dietary diversity and opportunistic infections

Statistical model	Adequate dietary diversity	Inadequate dietary diversity	
		OR (95% CI)	*p*-value
Model 1 (crude)	Ref.	4.20 (1.86 to 9.48)	0.001
Model 2	Ref.	3.93 (1.71 to 9.04)	0.001
Model 3	Ref.	3.03 (1.20 to 7.64)	0.019

Model 2 incorporated sociodemographic factors including age and income; in model 3, further adjustments were made for nutrition and clinical factors such as number of years on antiretroviral therapy, alcohol intake, household food security, Body Mass Index, anaemia status, and CD4 count. Model fit statistics for final adjusted model: −2 Log L = 164.8; Wald test = 64.5, *p* < 0.0001

The final model showing the likelihood of having OIs, and the covariates are presented in Table 4. With respect to the significant covariates, older respondents were approximately 8 times [AOR 7.9, (95% CI: 1.51 to 41.42), *p* = 0.014)] more likely to have OIs compared to their younger peers. Respondents with medium income were 70% less likely [AOR 0.30, (95% CI: 0.11 to 0.79), *p* = 0.015)] to have OIs compared to respondents with low income. Respondents on ART for > 5 years were 87% [AOR 0.13, (95% CI: 0.05 to 0.38), *p* < 0.001)] less likely to have OIs compared to those on ART for ≤ 1 year. Also, respondents on ART for > 1 to 5 years were 70% [AOR 0.30, (95% CI: 0.10 to 0.91), *p* = 0.033)] less likely to have OIs than respondents on ART for ≤ 1 year. Respondents in food-insecure households were also about 3 times [AOR 2.78, (95% CI: 1.05 to 7.37), *p* = 0.039)] more likely to have OIs compared to those in food-secured households. Respondents with low CD4 counts were about 4 times more likely [AOR 3.71, (95% CI: 1.34 to 10.3), *p* = 0.012)] to have OIs when compared to their peers with high CD4 counts.

**Table 4:** Final adjusted model showing the association between adequate dietary diversity and the occurrence of opportunistic infections

Variable	AOR (95% CI)	*p*-value
**Adequate dietary diversity**		
No (< 5 food groups)	3.03 (1.20 to 7.64)	***0.019***
Yes (> 4 food groups)	Ref.	
**Age (years)**		
18–35 (younger)	Ref.	
36–55 (middle-aged)	4.29 (1.03 to 18.38)	0.050
56+ (older)	7.90 (1.51 to 41.42)	***0.014***
**Monthly income**		
Low	Ref.	
Medium	0.30 (0.11 to 0.79)	***0.015***
High	0.37 (0.13 to 1.04)	0.060
**Years of antiretroviral therapy**		
≤ 1	Ref.	
> 1 to 5	0.30 (0.10 to 0.91)	***0.033***
> 5	0.13 (0.05 to 0.38)	***< 0.001***
**Alcohol intake (past week)**		
No	Ref.	
Yes	1.89 (0.58 to 6.19)	0.290
**Household food secured?**		
No	2.79 (1.05 to 7.37)	**0.039**
Yes	Ref.	
**Body Mass Index**		
Underweight	Ref.	
Normal	0.46 (0.12 to 1.74)	0.251
Overweight/obese	0.42 (0.11 to 1.61)	0.205
**Anaemia**		
No anaemia (≥ 10.5 g/dL)	Ref.	
Anaemic (< 10.5 g/dL)	2.16 (0.87 to 5.38)	0.098
**CD4 count**		
Low (< 500 cell/µl)	3.71 (1.34 to 10.3)	***0.012***
High (≥ 500 cell/µl)	Ref.	

Model fit statistics: 2 Log L = 164.8; Wald test = 64.5, *p* < 0.0001; Nagelkerke R^2^ = 0.37

AOR, adjusted Odds Ratio; Ref., Reference value.

## Discussion

The nutritional status of PLWHA is an important factor influencing disease progression and the effectiveness of ART. This study aimed to explore the dietary intake and food habits, the prevalence of OIs and other medical factors, and the association between dietary diversity and the occurrence of OIs among PLWHA on ART while controlling for confounding factors. The results revealed that a little more than half of the respondents had inadequate dietary diversity. The detrimental food habits included alcohol consumption, meal skipping and a low proportion of respondents with intake of group 4 (milk and milk products), group 5 (meat, poultry and fish) and group 10 (other fruits like mango, orange & watermelon) food groups. Also, about 1 in 7 respondents had at least one opportunistic infection, 3 in 5 had low CD4 counts, and 56% of respondents had been on ART for over 5 years. At the multivariable level, respondents with inadequate dietary diversity were three times more likely to have OIs when compared with their peers with adequate dietary diversity.

Findings from this study indicate that less than half of the respondents had an adequately diverse diet, which is similar to a study in Ghana [41]. However, this number is higher than reports from studies in Lawra Hospital, Cape Coast Teaching and the University of Cape Coast Hospitals [17, 42]. The percentages of adequate dietary diversities at these hospitals were 13.4% and 33.2%, respectively. In contrast, this prevalence was lower than that reported in a study conducted in six anonymized hospitals in Accra [43]. Differences in the timing/season of data collection could account for such variations. For instance, the study conducted in the six anonymized hospitals, collected data from October, coinciding with the postharvest period, when food prices were likely to be low and access high [43]. Additionally, interregional variations in dietary habits between respondents in studies from coastal Ghana and respondents in southern Ghana (where this study was conducted) could be a plausible explanation for the differences in the prevalence of dietary diversity [42, 43]. These interregional variations may include food taboos, where women in coastal Ghana tend to skip the consumption of specific foods when pregnant or differences in diets occasioned by an ecological zone induced dietary intake, where inhabitants of Ashanti region tend to consume more animal-sourced protein. This study also revealed that some participants (14.8%) consumed alcohol and that 1 in 3 skipped meals contrary to the WHO consolidated guidelines for adherence to ART [33]. This trend of unacceptable food habits among Ghanaian PWLHA was also identified in other studies [10, 15]. The findings also highlight a significant gap in the dietary intake of some participants, with a larger proportion of PLWHA not consuming relevant foods such as milk, milk products, meat, poultry, fish or other fruits. Although the WHO nutrient requirements for PLWHA technical report indicates that there is no scientific basis to recommend an increase in protein intake among PLWHA, it could however be detrimental for a large proportion of PLWHA to not consume protein-containing foods in adequate quantities [44, 45]. The high proportion of PLWHA having some detrimental food habits could be attributed to the data collection not coinciding with the post-harvest period. Skipping meals and alcohol intake were insignificant in further analyses, but previous studies have shown that such poor dietary habits can exacerbate specific micronutrient deficiencies, which have been associated with impaired immune function and increased susceptibility to infections [46, 47].

The study revealed that 13.4% of the respondents experienced OIs. This prevalence is relatively high and underscores the ongoing challenges in managing PLWHA, even those on ART. OIs are a major cause of morbidity and mortality among PLWHA, particularly in resource-limited settings. In Ghana, a study on OIs in the largest tertiary referral hospital reported a higher OI prevalence of 33.1% [21]. A systematic review and meta-analysis involving 6,163 PLWHA on ART in Ethiopia reported a much higher pooled prevalence of 44% [8]. These findings could be ascribed to the differences in the prevalence of HIV, access to ART and nutritional support. In resource-limited settings, such as Ethiopia, the high prevalence of HIV strains both human and logistical resources. This strain reduces the availability of ART and compromises the quality of nutritional support services due to increased demand [48, 49]. This evidence shows that access to ART, alongside nutritional support through counselling and food rations, is linked to a lower prevalence of opportunistic infections among people living with HIV/AIDS. This is largely because enhanced access to ART and nutritional support helps achieve a lower viral load and improved immune function, which mediate the reduction in infection rates among this population [48, 49].

The main finding of this study is the significant association between inadequate dietary diversity and the occurrence of OIs. After controlling for confounding factors, respondents with inadequate dietary diversity were found to be three times more likely to have OIs. In a particular study in Ethiopia with a similar finding, the time taken to develop OI was shorter for undernourished PLWHA than for those who were well nourished [9]. This direct association between inadequate dietary diversity and OIs can be justified in several ways. For instance, inadequate dietary intake contributes to immune dysfunction by impairing nutrition-acquired immune responses, thereby increasing the host’s susceptibility to infections [46]. Specifically, inadequate dietary diversity which contributes to malnutrition compromises the immune system by causing atrophy of the lymph nodes, thymus, and spleen, thereby attenuating cell-mediated immunity [50]. A deficiency in essential amino acids further affects the synthesis of proteins necessary for cytokine production, which is needed for the acute inflammatory response mediated by macrophages, lymphocytes, and other cells [51]. Collectively, these factors reduce the patient’s ability to fight and recover from such OIs.

Additional findings indicate that older respondents and patients in medium-income households were less likely to have OIs similar to the findings of a study in Mbagathi District Hospital, Kenya [52]. In contrast, patients with high CD4, on ART for 1 year or longer and in food-secure households were less likely to have OIs. The literature suggests that the loss of appetite and its accompanying poor dietary intake are notably associated with increasing age among the elderly [53]. As PLWHA get older, the effectiveness of mucosal barriers in the respiratory and gastrointestinal tracts, which are the first lines of defence against pathogens, decreases [54]. The thinning of these protective layers and reduced dietary intake increases the likelihood of the occurrence of OIs among older PLWHA. Regarding income, PLWHA in financially constrained households may delay seeking medical care due to transport costs and accessibility challenges [55]. These challenges can disrupt adherence to ART. As reduced ART adherence leads to weakened immune reconstitution, its direct and indirect consequences become more pronounced in this vulnerable group. Patients who remain on ART for a prolonged period often demonstrate better medication adherence [56]. ART adherence is essential for decreasing HIV-induced chronic inflammation and maintaining viral suppression [57]. This can be justified, as those in their early months may face more challenges with side effects which can reduce effectiveness and increase the risk of OIs. Finally, higher CD4 levels are associated with several immune-mediated functions that help minimize the risk of acquiring OIs. For instance, CD4 cells coordinate other components of the immune system, including cytotoxic CD8 cells, which kill virally infected cells, and B cells, which produce antibodies [58–60]. As such, lower CD4 cell counts breaks down this coordination, and the immune response to infections becomes weak and disorganized, making it easier for PLWHA to acquire OIs. CD4 cells also help the immune system develop memory responses to pathogens that it has previously encountered [59]. With fewer CD4 cells, the immune system loses this ability, and patients may suffer from recurrent infections or be unable to fight off previous exposures to pathogens.

### Implications for public health

Some programs are meant to provide nutritional support to PLWHA in Ghana. These programs include Nutritional Assessment, Counselling, and Support programs [61, 62]. The goal of these programs is to provide nutritional assessment, and nutrition counselling in the form of a feasible plan to improve nutritional status and therapeutic foods for moderately or severely undernourished PLWHA in Ghana [61, 62]. The findings of this present study warrant the need to strengthen nutritional counselling and support in HIV care, which could enhance the effectiveness of ART and reduce the burden of OIs. The findings also accentuate the importance of addressing not only dietary factors but other factors that may increase the risks of acquiring OIs, such as low CD4 counts, ART adherence and demographic factors such as age and income distribution. This can be achieved by exploring the possibility of providing alternate livelihoods to augment income and behaviour change communication (BCC) that encourages extended ART intake and regular CD4 check-ups. Dietary strategies such as the consumption of a varied and diverse diet should also be well communicated to PLWHA. Also, public health strategies, such as BCC, should also focus on reducing harmful behaviours such as alcohol consumption, skipping meals and promoting healthy lifestyle choices, with a particular emphasis on the double-burdened vulnerable group like aged PLWHA.

### Limitations and strengths

The use of a cross-sectional study design in this study prevented the ability to infer causality. Two factors limited the generalizability of this study including the use of a non-probability sampling technique and conducting this study in only one hospital. Additionally, the R^2^ value in the model fit statistics being 37% means that the study could not account for the other independent variables that explain the outcome variable. With the benefit of hindsight and considering the above, this study should have explored the in-depth reasons for such detrimental food habits, included other nutrition-sensitive variables such as water and sanitation, and a measure for ART adherence. Although the dietary diversity tool has been validated for women aged 15 to 49 years and may be used for other populations, caution should be exercised in interpreting and comparing this study’s findings to findings of other studies in which the original validated age group (women aged 15 to 49 years) are the respondents. However, the strengths of this study are the use of validated tools, the inclusion of a large sample size despite stigma-related challenges in recruiting PLWHA and robust statistical analyses.

## Conclusion

In conclusion, this study revealed that inadequate dietary diversity was a significant nutritional problem among PLWHA on ART in Kumasi South Hospital. The study also found a strong association between dietary diversity and the presence of OIs among PLWHA on ART. Despite a country-wide nutrition support program and increasing access to ART, there was a 3-fold association between inadequate dietary diversity and the risk of having OI among PLWHA at the study site. Hence, the nutrition support programs should be strengthened by using BCC to educate PLWHA on healthy dietary habits based on locally available diverse diets. Specific emphasis should be placed on the adequate consumption of foods in food group 4 i.e. milk and milk products, food group 5 i.e. meat, poultry and fish and food group 10 i.e. other fruits like mango, orange & watermelon food. Additionally, there is the need to improve household food insecurity and advocate for ART adherence and avoidance of alcohol consumption among PLWHA. These could decrease OIs and ultimately reduce HIV-related mortality.

## Electronic supplementary material

Below is the link to the electronic supplementary material.

Supplementary Material 1

## Data Availability

The datasets used and/or analysed during the current study are available from the corresponding author upon reasonable request. This is due to the stigma-prone subjects included in this study such as people living with HIV/AIDS. Also, the fact that data were collected from a single study site and the explicitly stated study period makes it inappropriate to publish such data openly. However, the dataset has been added to the manuscript for review purposes only.

## Abbreviations

AIDS: Acquired immune deficiency syndrome
ART: Antiretroviral therapy
BCC: Behaviour change communication
BMI: Body mass index
DDS: Dietary Diversity Score
DGT: Dolutegravir
EFV: Efavirenz
HFIAS: Household Food Insecurity Access Scale
HIV: Human immunodeficiency virus
NVP: Nevirapine
OIs: Opportunistic infections
PLWHA: People living with HIV/AIDS
