# Supplementary material for: Dietary diversity and opportunistic infections among adults living with human immunodeficiency virus on antiretroviral therapy in Kumasi metropolis; a facility-based cross-sectional study
Source: BMC Infect Dis. 2025 Jan 2;25:1. doi: 10.1186/s12879-024-10395-z (PMC11694380; doi:10.1186/s12879-024-10395-z)
Supplement: Supplementary file 1 — Supplementary Material 1 [file 12879_2024_10395_MOESM1_ESM.docx]

**KWAME NKRUMAH UNIVERSITY OF SCIENCE AND TECHNOLOGY, KUMASI**

**COLLEGE OF SCIENCE**

**DEPARTMENT OF BIOCHEMISTRY AND BIOTECHNOLOGY**

**A SURVEY ON DIETARY DIVERSITY, HOUSEHOLD FOOD INSECURITY,**

**NUTRITIONAL STATUS AND OPPORTUNISTIC INFECTIONS AMONG HIV INFECTED ADULTS IN THE KUMASI METROPOLIS**

**Dear participant,**

My name is ………………………………, and I am currently conducting a study on dietary diversity and opportunistic infections in Kumasi metropolis.

Today, I am here to complete surveys to understand the kinds of foods you consume, and your household food situation and some clinical challenges you may be having. In addition, I will be taking your height, weight and other body composition data, to evaluate your overall nutritional health status. I will also ask some questions about your household size, financial status and expenditure.

Your name will not be tied to this discussion and your contributions will remain anonymous. If you are uncomfortable with any of the questions, you may choose not to answer. However, your experience and opinions are very important to us, and we hope you will participate fully in this discussion. I expect the survey to take approximately 45 minutes.

**Do you agree to participate in this discussion?**

1. Yes, I agree [ ] ***(If yes, continue the interview)***

1. No, I do not want to take part [ ] ***(If no, end the interview)***

|  |
| --- |

**c. Your Participant Code is**

**SECTION A: GENERAL DEMOGRAPHIC INFORMATION**

1. **Gender:**  Male [ ] Female [ ]
2. **Age (years)**: _____________
3. Which region do you come from? **_**_________________
4. What is your current region of residence? ____________________
5. **Level of formal education completed**: None [ ] Basic [ ] Junior high [ ] Secondary+ [ ]
6. **Religious Affiliation:** Christian [ ] Islam [ ] Traditionalist [ ] Other: ______________
7. **Marital status:** 1. Married [ ] 2. Single, never married [ ] 3. Widowed [ ] 4. Divorced/Separated [ ] 4. Co-habiting [ ]
8. **Main occupation** 1. Farming (crops and farm animal rearing) [ ] 2. Salaried work [ ] 3. Artisan/Construction/ Vocational work [ ] 4. Trading [ ] 5. Agro-processing [ ] 6.
9. Unemployed [ ] 7. Housewife [ ] Others [ ]: *specify _____________*
10. **Who is your employer?** 1. Government [ ] 2. Private Institution [ ] 3. Self-Employed [ ] 4. Non-Profit Organization [ ] 5. Other *_____________*
11. **Ethnic affiliation:** Akan [ ] Ewe [ ] Ga Adamgbe [ ] Mole Dagbani [ ] Guan [ ] Non-Ghanaian [ ]
12. **Household size**: Total *_________ ; Males___________; Females__________*
13. **Is the respondent the household head/breadwinner**? Yes [ ] No [ ]
14. **If No, what is this respondent’s relationship with the household head/breadwinner?** Spouse of household head [ ] Male child of household head [ ] Female child of household head [ ] External family member [ ] A non-family member [ ]
15. What is the Gender of the household head/breadwinner? Male [ ] Female [ ]
16. Age of household head/breadwinner _______________ years
17. Household members who are below 18 years? ________________
18. Household members who are above 60 years? ________________
19. Household members who are dependants? _______________
20. Household members who are infirmed/diseased/disabled? ______________
21. Estimated monthly household income? Gh₵ *________________*
22. Estimated monthly household expenditure? Gh₵ *_________________*
23. Have you ever had any form of support (i.e. financial/food/education) Yes [ ] No[ ]
24. If yes, from which Persons/Institution/Organisation? *_____________*

**SECTION B: NUTRITIONAL STATUS (FOOD INTAKE & ANTHROPOMETRIC MEASUREMENTS OF PARTICIPANTS)**

1. Height (cm): ……………………………………………..
2. Weight (kg) ……………………………………………..
3. BMI (kg/m^2^): …………………….....................................
4. Total Body Fat: …………………………………………..
5. Visceral Fat: ……………………………………………...
6. Muscle Mass: ……………………………………………..
7. Resting Energy Expenditure (REE) …………………………………………….
8. Body Age: ………………………………………………………………………
9. Level of physical activity (1-5) ………………………………………………….
10. Estimated Daily Energy Requirement (kcal)…………………………………….
11. Estimated Daily Energy Consumption (kcal)…………………………………….

**SECTION C: NUTRITION AND FOOD CONSUMPTION HABITS AND ATTITUDES**

1. **Frequency of meals per day?** 1 [ ] 2 [ ] 3 [ ] ≥4 [ ]
2. **Do you skip meals?** Yes [ ] No [ ] If yes, how often? ______________
3. **How often do you eat from home?** 1. Always [ ] Mostly(≥2/day) [ ] Sometimes(≤5/wk)[ ] Rarely[] Not at all [ ]
4. **Which of these meals do you often take from home?** Breakfast [ ] Lunch[ ] Supper [ ] Snacks [ ] N/A[ ]
5. **If you consume foods made outside home, what is your reason?**

Unable to cook for self [ ] 2. Unable to afford home-made meals [ ] 3.Think commercially-made meals are cheaper[ ] 4.Enjoy outside meals more[ ] 5. Other reason(s)[ ]._________

1. **Do you consume any of the following foods? Indicate frequency of intake in the past 1 week**

Alcohol/Alcoholic beverages [ ]______________ Canned/packaged foods [ ]_____________ Sweetened snacks/beverages [ ]________________ Salted foods (e.g. koobi) [ ]____________ Baked foods(e.g. pie, cakes) [ ]________________ Polished/processed foods [ ] ____________

1. **Do** **you** **smoke**? Yes [ ] No[ ] If yes, how frequent in a week? _____________________

**SECTION D: CLINICAL FACTORS**

1. Type of antiretroviral therapy (ART) ___________________________________
2. For how long has he/she been on ART? _____________________________
3. Presence of opportunistic infections? Yes [ ] No [ ]
4. Type of opportunistic infection? ___________________________
5. Most recent CD4 count*: _____________________________*
6. Haemoglobin level ___________________________

**SECTION E: DIETARY DIVERSITY ASSESSMENT**

*Now I would like to ask you some yes-or-no questions about foods and drinks that you consumed yesterday during the day or night, whether you had it at home or somewhere else.*

*First, I would like you to think about yesterday, from the time you woke up through the night.*

*Think to yourself about the first thing you ate or drank after you woke up in the morning.*

**24-Hour recall dietary assessment**

| **Food/Beverage Items (List all foods and beverages for every meal and snack during the 24hour period)** | **Portion sizes (how many ladles, pieces, balls, slices, sachets teaspoons/tablespoons)** | **How was it Prepared (boiled, steamed, roasted, grilled, fried)?** | **Was anything added to it?** |
| --- | --- | --- | --- |
| **Breakfast:** |  |  |  |
|  |  |  |  |
|  |  |  |  |
|  |  |  |  |
|  |  |  |  |
| **Morning snack:** |  |  |  |
|  |  |  |  |
|  |  |  |  |
| **Lunch:** |  |  |  |
|  |  |  |  |
|  |  |  |  |
|  |  |  |  |
|  |  |  |  |
| **Afternoon snack:** |  |  |  |
|  |  |  |  |
|  |  |  |  |
| **Supper/Dinner:** |  |  |  |
|  |  |  |  |
|  |  |  |  |
|  |  |  |  |
|  |  |  |  |
| **Night snack:** |  |  |  |
|  |  |  |  |
|  |  |  |  |
|  |  |  |  |

*Think about where you were when you had any meal. And any food or drink you may have had from morning to evening or late-night. Also, any other snacks or drinks you may have had between meals throughout the day and night. I am interested in whether you had the food items I will mention even if they were combined with other foods. Please, listen to the list of foods and drinks, and if you ate or drank* ***ANY ONE OF THEM****, say yes.*

|  | **GROUP 1 – GRAINS, WHITE ROOTS AND TUBERS AND PLANTAINS** | **(Circle**  **answer)** |
| --- | --- | --- |
| 1 | Bread, rice, waakye, jollof, fried rice, omutuo, rice porridge noodles/pasta, or sorghum? | YES or NO |
| 2 | Kenkey, banku, tou zaafi, Hausa koko, akple, roasted maize, boiled maize, or tom brown? | YES or NO |
| 3 | Fufu, gari, kokonte, cassava, yam, cocoyam, plantain, or sweet potato? | YES or NO |
|  | **GROUP 2 – PULSES (BEANS, PEAS AND LENTILS)** |  |
| 4 | Beans, bambara beans, peas, lentils, hummus, tofu, tempeh | YES or NO |
|  | **GROUP 3 – NUTS AND SEEDS** |  |
| 5 | Groundnut/peanut, cashew, walnut, Baobab seeds, chia seeds, flaxseed | YES or NO |
| 6 | Groundnuts, kuli kuli, groundnut paste, groundnut soup, agushi stew, neri soup, or cashews? | YES or NO |
|  | **GROUP 4 – MILK AND MILK PRODUCTS** |  |
| 7 | Fresh milk, tin milk, or powdered milk? | YES or NO |
| 8 | Cheese curds, wagashi, Brukina or yogurt? | YES or NO |
|  | **GROUP 5 – MEAT, POULTRY AND FISH** |  |
| 9 | Sausages or corned beef? | YES or NO |
| 10 | Gizzard, heart, kidney, liver? | YES or NO |
| 11 | Beef, goat, sheep, pork, glasscutter, rabbit, or bush meat? | YES or NO |
| 12 | Chicken, duck, goose or Guinea fowl? | YES or NO |
| 13 | Fresh, frozen or dried fish, koobi, anchovies, smoked herring, crab, or shrimp? | YES or NO |
|  | **GROUP 6 – EGGS** |  |
| 14 | Eggs from poultry or any other bed? | YES or NO |
|  | **GROUP 7 – DARK GREEN LEAFY VEGETABLES** |  |
| 15 | Cocoyam leaves, amaranth leaves, ademe, ayoyo, sweet potato leaves, cassava leaves, or bokoboko? | YES or NO |
| 16 | Baobab leaves, cowpea leaves, roselle leaves or bra, kenaf, African eggplant leaves, or West India nettle, spinach, broccoli? | YES or NO |
|  | **GROUP 8 –VITAMIN A-RICH FRUITS AND VEGETABLES** |  |
| 17 | Carrots, or sweet potatoes that are orange inside. | YES or NO |
| 18 | Ripe mango, ripe papaya, or African star apple? | YES or NO |
|  | **GROUP 9 – OTHER VEGETABLES** |  |
| 19 | Tomatoes, okro, garden eggs, sponge gourd, cabbage, or mushrooms? | YES or NO |
| 20 | Beets, cauliflower, celery, sweet green pepper, or cucumber? | YES or NO |
|  | **GROUP 10 – OTHER FRUITS** | YES or NO |
| 21 | Orange or tangerine? |  |
| 22 | Banana, pineapple, avocado pear, watermelon, apple, grapes, berries or guava? | YES or NO |
| 23 | Soursop, coconut flesh, velvet tamarind, baobab, ebony fruit, or shea fruits? | YES or NO |

**SECTION F: HOUSEHOLD FOOD INSECURITY ASSESSMENT**

***In this section, I would like you to respond to these questions. As much as applicable to you, your sincere response will help achieve the goal of this study.***

| **No.** | **QUESTION** | **RESPONSE OPTIONS** | **SCORE**  **(0 to 3)** |
| --- | --- | --- | --- |
| **1** | In the past four weeks, did you worry that your household would not have enough food? | 0 = No (skip to Q2)  1=Yes |  |
| **1.a** | How often did this happen? | 1 = Rarely (once or twice in the past four weeks)  2 = Sometimes (three to ten times in the past four weeks)  3 = Often (more than ten times in the past four weeks) | **……** |
| **2** | In the past four weeks, were you or any household member not able to eat the kinds of foods you preferred because of a lack of resources? | 0 = No (skip to Q3)  1=Yes |  |
| **2.a** | How often did this happen? | 1. = Rarely (once or twice in the past four weeks) 2. = Sometimes (three to ten times in the past four weeks) 3. = Often (more than ten times in the past four weeks) | **……** |
| **3** | In the past four weeks, did you or any household member have to eat a limited variety of foods due to a lack of resources? | 1. = No (skip to Q4) 2. = Yes |  |
| **3.a** | How often did this happen? | 1. = Rarely (once or twice in the past four weeks) 2. = Sometimes (three to ten times in the past four weeks) 3. = Often (more than ten times in the past four weeks) | **……** |
| **4** | In the past four weeks, did you or any household member have to eat some foods that you really did not want to eat because of a lack of resources to | 1. = No (skip to Q5) 2. = Yes |  |
| **4.a** | How often did this happen? | 1. = Rarely (once or twice in the past four weeks) 2. = Sometimes (three to ten times in the past four weeks) 3. = Often (more than ten times in the past four weeks) | **……** |
| **5** | In the past four weeks, did you or any household member have to eat a smaller meal than you felt you needed because there was not enough food? | 1. = No (skip to Q6) 2. = Yes |  |
| **5.a** | How often did this happen? | 1. = Rarely (once or twice in the past four weeks) 2. = Sometimes (three to ten times in the past four weeks) 3. = Often (more than ten times in the past four weeks) | **……** |
| **6** | In the past four weeks, did you or any other household member have to eat fewer meals in a day because there was not enough food? | 1. = No (skip to Q7) 2. = Yes |  |
| **6.a** | How often did this happen? | 1. = Rarely (once or twice in the past four weeks) 2. = Sometimes (three to ten times in the past four weeks) 3. = Often (more than ten times in the past four weeks) | **……** |
| **7** | In the past four weeks, was there ever no food to eat of any kind in your household because of lack of resources to get food? | 1. = No (skip to Q8) 2. = Yes |  |
| **7.a** | How often did this happen? | 1. = Rarely (once or twice in the past four weeks) 2. = Sometimes (three to ten times in the past four weeks) 3. = Often (more than ten times in the past four weeks) | **……** |
| **8** | In the past four weeks, did you or any household member go to sleep at night hungry because there was not enough food? | 1. = No (skip to Q9) 2. = Yes |  |
| **8.a** | How often did this happen? | 1. = Rarely (once or twice in the past four weeks) 2. = Sometimes (three to ten times in the past four weeks) 3. = Often (more than ten times in the past four weeks) | **……** |
| **9** | In the past four weeks, did you or any household member go a whole day and night without eating anything because there was not enough food? | 1. = No (questionnaire is finished) 2. = Yes |  |
| **9.a** | How often did this happen? | 1. = Rarely (once or twice in the past four weeks) 2. = Sometimes (three to ten times in the past four weeks) 3. = Often (more than ten times in the past four weeks) | **……** |
| **Total score (0 to 27) =** | | | |
